# Relating household entomological measures to individual malaria risk

**DOI:** 10.1186/s12936-025-05504-5

**Published:** 2025-08-14

**Authors:** Max McClure, Emmanuel Arinaitwe, Moses R. Kamya, Philip J. Rosenthal, Joaniter Nankabirwa, Maxwell Kilama, Alex Musiime, Grant Dorsey, Bryan Greenhouse, Isabel Rodriguez-Barraquer

**Affiliations:** 1 Department of Medicine, San Francisco General Hospital, University of California, San Francisco, San Francisco, USA; 2 Infectious Diseases Research Collaboration, Kampala, Uganda; 3 Makerere University College of Health Sciences, Kampala, Uganda; 4 National Malaria Control Division, Ministry of Health, Kampala, Uganda; 5 Chan Zuckerberg Biohub, San Francisco, CA USA

## Abstract

**Background:**

The gold standard measure of malaria exposure is the entomological inoculation rate (EIR), or the number of infectious bites an individual receives over a given period. Nevertheless, it is unclear whether EIR measured in the households of individuals reflects heterogeneity in those individuals’ infection risk.

**Methods:**

To investigate this relationship, this study used data collected from a cohort of 439 children aged 0.5–5 years in 239 households from 2011–2016 in three Ugandan districts: low-EIR Jinja, intermediate-EIR Kanungu and high-EIR Tororo. Participants underwent passive and quarterly active surveillance for clinical malaria, defined as fever with positive thick blood smear. Monthly vector densities and sporozoite rates in participating households were estimated using CDC light traps. The association between spatiotemporally smoothed household log_2_-transformed EIR and individual malaria incidence was assessed using Poisson generalized additive mixed effects models.

**Results:**

Comparison across sites suggested an increasing relationship between average EIR and malaria incidence. Within-site relationships, however, varied by site, with a positive association in Kanungu (incidence rate ratio [IRR] 1.09, 95% credible interval 1.04–1.14) but none in Jinja (1.02, 0.774–1.26) or Tororo (1.02, 0.986–1.06).

**Conclusions:**

These results show the relationship between measured EIR and malaria incidence may depend on site-specific transmission dynamics and be strongest at intermediate EIR, while underscoring the challenges of using household-level measures of exposure.

**Supplementary Information:**

The online version contains supplementary material available at 10.1186/s12936-025-05504-5.

## Background

Anopheline mosquitoes transmit malaria to humans, and exposure to *Plasmodium*-carrying mosquitoes corresponds to human malaria risk. The World Health Organization (WHO) guidelines recommend surveillance of entomological proxies for transmission as a component of integrated vector management programs [1, 2]. However, current understanding of the quantitative relationship between mosquito exposure and human infection risk is surprisingly limited.

The gold standard entomological measure of malaria exposure is the entomological inoculation rate (EIR), or the number of infectious bites received by an individual in a given time period [2]. EIR is typically calculated as the product of the vector density as determined by mosquito captures—an approximation of host-seeking vector density—and the proportion of tested mosquitoes positive for sporozoites [3]. EIR has a positive relationship to parasite prevalence [4] that has been validated with real-world data at community scales [5, 6], and an association with the incidence of blood stage infections down to the village or neighbourhood level [7, 8], but it is unclear if it is able to capture heterogeneity in transmission driving incidence at smaller scales such as the household or the individual. Factors potentially obscuring such an association include measurement error and uncaptured spatiotemporal variability in entomological indices, as well as behavioural heterogeneity between vector species, and behavioural and immunologic heterogeneity among human hosts.

To better assess the association between EIR and individual malaria incidence, this study analysed data from completed longitudinal cohort studies that included passive and active clinical surveillance of participants and paired household-level entomological measurements at sites with varying transmission. Flexible spatiotemporal models were used to smooth crude entomological measures and allow for nonlinear associations between exposure and human disease risk, with the goal of more precisely addressing concerns about uncertainty and spatiotemporal variability in entomological data. These cohorts were well-suited to assess associations between mosquito exposure and clinical outcomes given their concurrent entomological and clinical data collection across a range of transmission intensities, a focus on children (with lower rates of acquired immunity compared to adults), and relatively stable transmission during the study period.

## Methods

### Study location

The original Program for Resistance, Immunology, Surveillance and Modeling of Malaria (PRISM 1) cohort studies were conducted from 2011–2017 in three Ugandan subcounties representing a spectrum of malaria transmission settings: Walukuba, Jinja District, a peri-urban area near the northern shore of Lake Victoria with the lowest transmission; Kihihi, Kanungu District, a rural area in the southwest near the country’s border with the Democratic Republic of the Congo with relatively moderate transmission; and Nagongera, Tororo District, a rural area in the southeast near the country’s border with Kenya with the highest transmission.

*Plasmodium falciparum* is the dominant malaria parasite throughout Uganda. In the nationally representative 2009 Uganda Malaria Indicator Survey, which speciated *Plasmodium* parasites based on examination of thick smears, 99% of infected children carried *P. falciparum* [9].

During the data collection period, long-lasting insecticidal nets (LLIN) were distributed in Jinja in November 2013, Kanungu in June 2014, and Tororo in November 2013; study participants were provided access to an LLIN at enrolment (regardless of enrolment timing). LLINs were also provided any time a participant came to the clinic and said they no longer had access to one. For this analysis, in Tororo only, any data collected after a participating household underwent indoor residual spraying (IRS) was excluded. IRS was administered in Tororo district after December 2014, but was not implemented in Jinja or Kanungu.

### Study design and data collection

This study analysed data from existing cohorts aiming to provide a comprehensive description of malaria epidemiology in Uganda. The analysis used all available data from the parent studies, the study protocol for which is described elsewhere [10, 11]. Briefly, all households within each subcounty were enumerated. 100 households containing at least one resident 0.5–10 years of age and one adult resident were then randomly selected from the enumerated households in each subcounty and enrolled in August and September 2011 (with additional households enrolled in August and September 2013 to replace households that withdrew). All children in each household between 0.5 and 10 years of age that met study eligibility criteria were enrolled. Household latitude and longitude were mapped using handheld GPS and projected to UTM zone 36 coordinates. Individual enrolment was dynamic over the course of the study—children from participating households joined the study as they became eligible and left as they aged out.

Routine clinical visits were conducted every 3 months and included a standardized clinical assessment and collection of thick blood smears. Participants were encouraged to present to dedicated study clinics (one per site) for evaluation of any medical needs outside routine visits: if participants were febrile at the time of evaluation or reported fever within the past 24 h, thick blood smears were obtained. Malaria was defined as a fever (tympanic temperature ≥ 38 °C or reported fever within the past 24 h) with a thick blood smear positive for malaria parasites [10]. Participants diagnosed with malaria were treated according to national treatment guidelines (artemether-lumefantrine for uncomplicated malaria, quinine for complicated or recurrent malaria).

The PRISM 1 cohort study enrolled an equal number of households in each subcounty, comprising 454 participants in Jinja, 478 in Kanungu, and 470 in Tororo. To reduce the impact of immunity and host factors that may reduce the probability of an infection leading to clinical malaria, this analysis excluded members of the cohort who were older than 5 years of age or had documented sickle cell trait or disease, resulting in a sample of 439 participants from 239 households.

Mosquito collections were conducted one night a month using CDC light traps (CDC LT) set from 7 pm to 7am 1 m above the floor at the foot of the bed in one bedroom of each participating household. All mosquito species were identified morphologically. Based on PCR identification of a subset of CDC LT captures at these study sites from 2011–2016, during the study period *Anopheles arabiensis* was dominant in Jinja [54.3%, with 34.1% *Anopheles gambiae *sensu stricto (*s.s.)* and 5.3% *Anopheles funestus*), *An. gambiae s.s.* was dominant in Kanungu (98.1%, with 0.7% *An. funestus* and 0.6% *An. arabiensis*), and Tororo (67.5%, with 21.6% *An. arabiensis* and 10.1% *An. funestus*) [11].

A random subset of mosquitoes from each capture (maximum 50 per collection) was stored on desiccant and tested for sporozoites using an enzyme-linked immunosorbent assay (ELISA) method [12, 13]. Results from light trap collections at these sites were previously shown to be strongly correlated with contemporaneous human-landing catches, which were only available from 2011–2012 [13].

### Statistical analyses

The objective of this analysis was to characterize the association between entomological surveillance data and incidence of malaria. First, multiple spatiotemporal models of entomological data were developed to obtain smoothed estimates of household EIR. The predicted EIRs from the best-fitting of these models were then used to assess the relationship between EIR and malaria incidence.

### Modelling EIR over time and space

To model total mosquito counts for the region’s two major vectors combined, *An. gambiae *sensu lato (*s.l.)* and *An. funestus* group, negative binomial spatiotemporal generalized additive models (GAMs) were fit for each site, using thin plate splines for temporal smooths and either low rank gaussian process smooths with a power exponential correlation function or thin plate splines to describe the interaction of household projected coordinates. Additional model types were considered as detailed in the supplementary materials.

A similar process was followed to model *Anopheles*-wide sporozoite rates for all mosquitoes that underwent ELISA: these were fit as spatiotemporal binomial GAMs. Independent spatial and temporal smooths were used as described above.

In both cases, models without concerning over- or under-dispersion or heteroscedasticity were compared by Akaike information criterion (AIC). For each best-fitting model type, models specified with spatial smooths only, temporal smooths only, and spatial and temporal smooths were then compared by AIC. The best-fitting models after this step were used to generate daily predicted log_2_-transformed annualized EIR (aEIR), calculated as the product of the predicted sporozoite rate and predicted vector count multiplied by 365 (subsequently referred to as modeled aEIR).

All GAMs were fit with the mgcv package in R [14]. Residual diagnostics to assess concerning under- or over-dispersion, quantile deviations or influential outliers were performed using the DHARMa package [15].

### Modelling the association between incidence of malaria and EIR

Incident malaria was chosen as an admittedly imperfect proxy for incident infection: prior studies have demonstrated that the majority of asymptomatic infections in young children progress to symptomatic malaria, so this analysis was limited to children under 5 years of age [16].

The relationship between an individual’s household level aEIRs (log_2_-transformed as described above) and their daily malaria incidence was modelled for each site with a Poisson mixed effects GAM (GAMM) using a thin plate spline smooth in the mgcv package in R. The 14 days following an episode of malaria were excluded from analysis to account for the prophylactic effect of antimalarial treatment. aEIR was lagged by 14 days to account for the *P. falciparum* intrinsic incubation period; 28-day lags were also evaluated and yielded qualitatively similar results. All models controlled for participant age using a thin plate spline basis and included individual and household IDs as random effects, such that incidence models took the following form (where *i* represents date, *j* household, *k* an individual participant, *µ*_*j*_ and *γ*_*k*_ the household and individual random effects, and *Y*_*i,j,k*_ a random variable:

1 $$log\left( {case \, count_{i,j,k} } \right) \, = \, f\left( {log_{2} \left( {14d - lagged \, aEIR_{i,j} } \right)} \right) \, + \, f\left( {age_{k} } \right) \, + \, \mu_{j} + g_{k} , \, Y_{i,j,k} \sim Poi\left( {case \, count_{i,j,k} } \right)$$

To generate interpretable incidence rate ratios (IRRs), model fitting was repeated treating the association between log_2_-transformed modelled aEIR and incidence as linear on the log scale.

To account for uncertainty in entomological parameter estimation, prediction intervals were generated for the entomological GAMs by drawing 1000 samples from the posterior of the fitted values of the models using the gratia package in R [17]. Binomial model samples were weighted according to the number of mosquitoes collected per household over the study period. The GAMMs with the covariates listed above were then refit to these draws and an additional 1000 samples were drawn from the posterior of the expected value of the model responses, which were then used to calculate aEIR as described above. Reported smooths and IRRs reflect the means and 2.5% and 97.5% quantiles of the pooled results and are adjusted for all listed covariates unless otherwise specified.

While the main analysis was based on aEIRs derived from the best fitting models of mosquito counts and sporozoite rates, the association between malaria incidence and crude aEIRs—defined as the products of vector count and sporozoite rate for each capture session—and modelled aEIRs that omitted either spatial or temporal smooths was also assessed. The fit of models incorporating different estimates of aEIR were compared using the AIC. For these comparisons, the expected responses of entomological models were used rather than the pooled prediction intervals described above.

## Results

### Study population

**Fig. 1:**
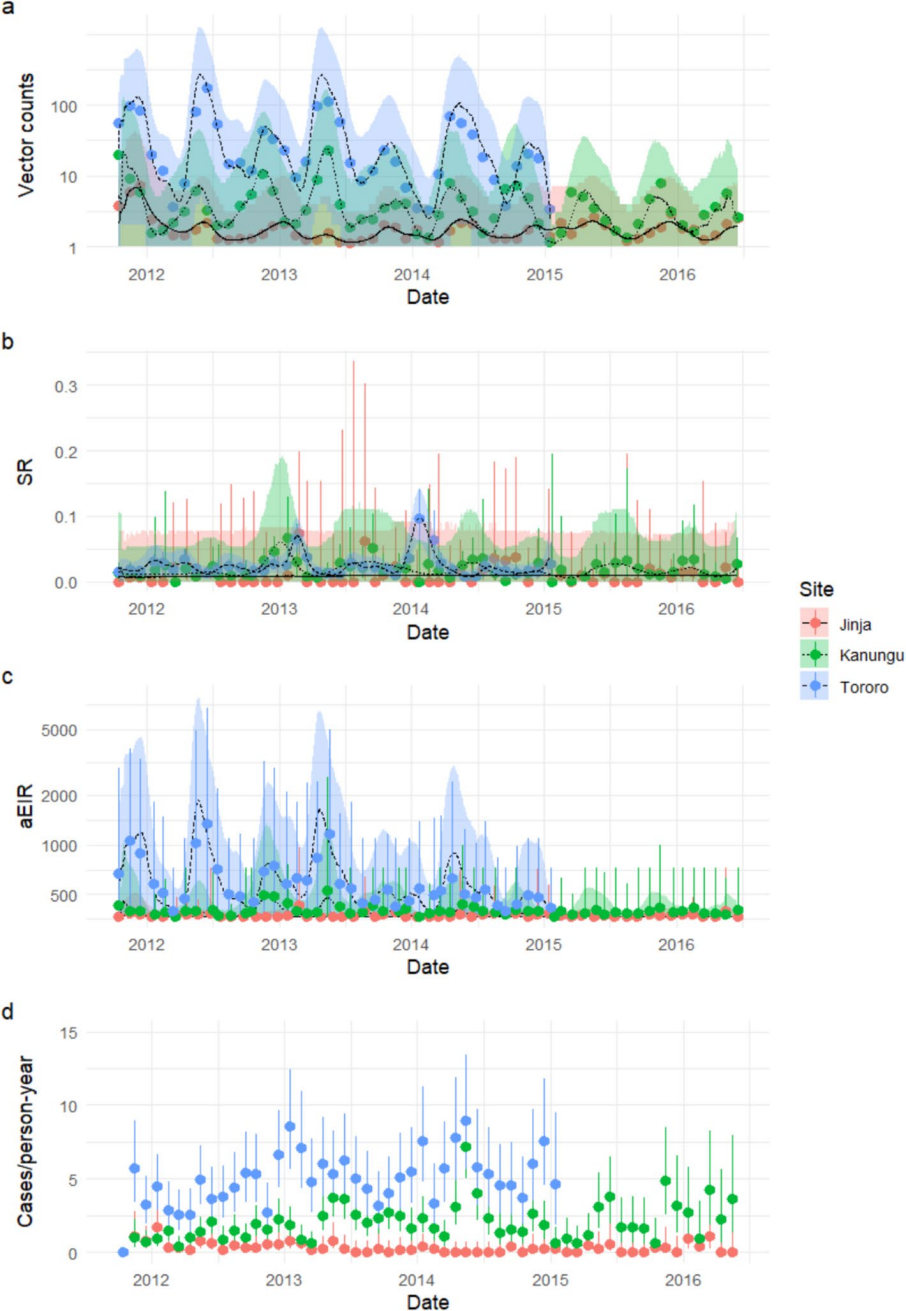
Monthly vector count, sporozoite rate, annualized entomological inoculation rate (aEIR) and malaria incidence (cases per person-year) by site for the period 2012–2016. Points with whiskers represent mean monthly crude data and associated uncertainty, while lines with confidence bands represent mean spatiotemporal model outputs and associated uncertainty. For vector counts, whiskers show 95% confidence intervals for summed *Anopheles gambiae* s.l. and *Anopheles funestus* s.l. counts modeled as a Poisson process. For sporozoite rate and malaria incidence, whiskers show 95% confidence intervals from the exact binomial test. For aEIR, whiskers represent 2.5% and 97.5% quantiles of collection-level aEIRs. Confidence bands for all GAM outputs represent the 2.5% and 97.5% quantiles of pooled draws as described in the text

Characteristics of the study population are shown in Table 1. Participants were followed for a median of 650 days (interquartile range [IQR] 324–1078), during which the median number of cases per person-year was 0 (IQR 0–0.481) in Jinja, 1.14 (0–2.56) in Kanungu, and 4.07 (2.14–7.41) in Tororo. Monthly trends in malaria incidence are shown in Fig. 1D, demonstrating two annual peaks at each site. The highest incidences were consistently observed in Tororo, followed by Kanungu and then Jinja.

**Table 1:** Characteristics of study participants

	Jinja	Kanungu	Tororo	Overall
No. participants	145	169	125	439
No. households	82	84	73	239
Age (IQR)	2.1 (1, 3.6)	2.1 (1.3, 3.4)	2.3 (1.1, 4)	2.2 (1.1, 3.6)
% Male	48.3	49.7	54.4	50.6
Median days followed (IQR)	593 (346, 1079)	755 (490, 1151)	589 (246, 993)	651 (324.5, 1079)
Median malaria cases per person (IQR)	0 (0, 1)	2 (0, 5)	5 (1, 9)	1 (0, 5)
Median malaria cases per person-year (IQR)	0 (0, 0.481)	1.14 (0, 2.56)	4.07 (2.14, 7.41)	0.96 (0, 3.43)
Median proportion of nights using LLIN (IQR)	1 (1, 1)	1 (1, 1)	1 (1, 1)	1 (1, 1)

Age refers to age in years at time of enrolment

### Variation in EIR over time and space

Monthly entomological measures over the study period are summarized in Table 2, with monthly subcounty-wide trends in *Anopheles* counts, sporozoite rate, crude aEIR and malaria incidence among cohort participants shown in Fig. 1. As expected, the highest vector densities and sporozoite rates were recorded in Tororo, the second-highest in Kanungu, and the lowest in Jinja. Site-specific aEIRs calculated over the course of the study consequently followed the same pattern: 233 in Tororo, 20.3 in Kanungu, and 2.72 in Jinja.

**Table 2:** Summary statistics for monthly entomological measures

	Jinja			Kanungu			Tororo			Overall		
	Collection median (IQR)	HH median (IQR)	Site	Collection median (IQR)	HH median (IQR)	Site	Collection median (IQR)	HH median (IQR)	Site	Collection median (IQR)	HH median (IQR)	Site
Vector count	0 (0,1)	0.429 (0.281,1)	0.89	0 (0,2)	1.4 (0.482,4.58)	3.41	10 (2, 39)	28.6 (19,41.8)	33.5	0 (0, 4)	2.51 (0.441, 19.8)	10.5
SR	0 (0,0)	0 (0,0.00147)	0.00837	0 (0,0)	0.00915 (0,0.0222)	0.0163	0 (0, 0)	0.0172 (0.0138,0.0236)	0.019	0 (0, 0)	0.0111 (0, 0.0208)	0.018
aEIR	0 (0,0)	0 (0,3.2)	2.72	0 (0,0)	8.15 (0,27)	20.3	0 (0,0)	178 (108,307)	233	0 (0, 0)	11.1 (0, 111)	69.3
No. collections	NA	57 (33,57)	5210	NA	56 (56,57)	5410	NA	40 (39,40)	3860	NA	45 (39,57)	14,500

To generate aggregated measures, collection-level measurements were summed by household (“HH”) or site and divided by number of collections. Collection-level measures, which constituted the response variable of the smoothed entomological models used in the final analysis, are included to illustrate the zero inflation described in the main text

The number of mosquitoes collected during any single collection was consistently low in Jinja and varied little over the study: the median number of mosquitoes collected per household (averaged over the entire study period) was 0.429 (IQR 0.281–1), but the median number at any given collection was 0 (IQR 0–1), and 0 mosquitoes were collected in 3697/5212 (70.9%) collections. Counts varied more widely at the two other sites: in Kanungu, the median per household was 1.4 (0.482–4.58) and median per collection 0 (0–2), with 0 mosquitoes collected in 2977/5414 (55.0%) collections; in Tororo, the median per household was 28.6 (19–41.8) and median per collection 10 (2–39), with 0 mosquitoes collected in 656/3858 (17.0%) collections.

Median sporozoite rates by household were less variable: 0 (0–0) in Jinja, 0.00915 (0–0.0222) in Kanungu, and 0.0172 (0.0138–0.0236) in Tororo. Median sporozoite rate per collection was 0 (0–0) for all sites, as sporozoite rates were equal to 0 in the majority of captures in all three: 5175/5212 (99.3%) of captures in Jinja, 5209/5414 (96.2%) in Kanungu, and 2955/3858 (76.6%) in Tororo.

The observed sparseness and variability prompted us to consider multiple spatiotemporally smoothed models of vector counts and sporozoite rates. For vector counts, the best-fitting models explained a moderate percentage of the deviance at all sites (Jinja: 45.0; Kanungu: 60.6; Tororo: 57.4) (Supplementary Table 1). For sporozoite rates, the best-fitting models explained a small percentage of the deviance, particularly in Jinja (Jinja: 1.83; Kanungu: 11.1; Tororo: 11.8) (Supplementary Table 1).

Predictions generated from the best vector density and sporozoite rate models are overlaid on Fig. 1A–C. At all sites, biannual peaks in vector counts were observed, corresponding roughly to the March–May and August–October rainy seasons. Increases in sporozoite rates corresponded to decreases in vector count, as expected in an aging mosquito population. Vector counts were higher at the eastern border of the study site in Jinja, nearer to Lake Victoria, and at the northern border in Kanungu, where altitude was lower, but were patchy in Tororo. Sporozoite rates were higher in the southwest in Jinja, patchy in Kanungu, and lacked notable spatial structure in Tororo (Supplementary Fig. 1). Temporal and spatial trends in EIR were similar to those for vector counts.

### Association between entomological metrics and malaria incidence

**Fig. 2:**
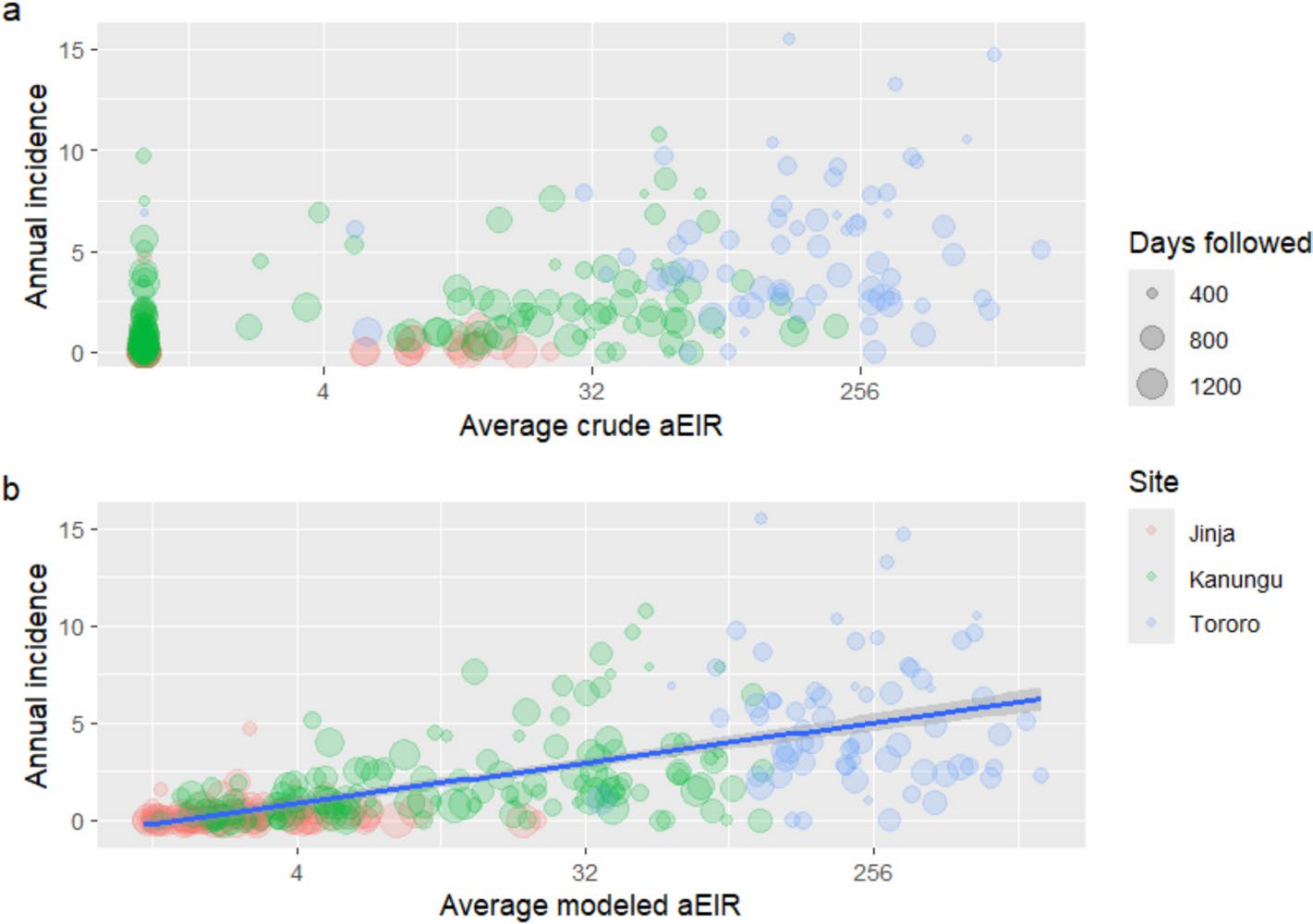
Association between household mean crude (a) or modeled (b) annualized entomological inoculation rate (aEIR) and individuals’ annual malaria incidence experienced over the course of the study, grouped by site. Plots are restricted to individuals followed for at least 365 days. Point sizes represent the duration of follow-up time in days. In panel b, a line of best fit with 95% confidence intervals is overlaid across all sites

The association between aEIR and malaria incidence was evaluated next. Figure 2 shows the association between average household aEIR and the average incidence of malaria experienced by individuals over the course of the study, for both crude (2A) and modeled (2B) aEIRs. Although analysing data from the three sites together suggests a positive relationship between aEIR and malaria incidence, much of this association might be attributed to between-site differences, since both aEIR and incidence were lowest in Jinja, moderate in Kanungu and highest in Tororo.

**Fig. 3:**
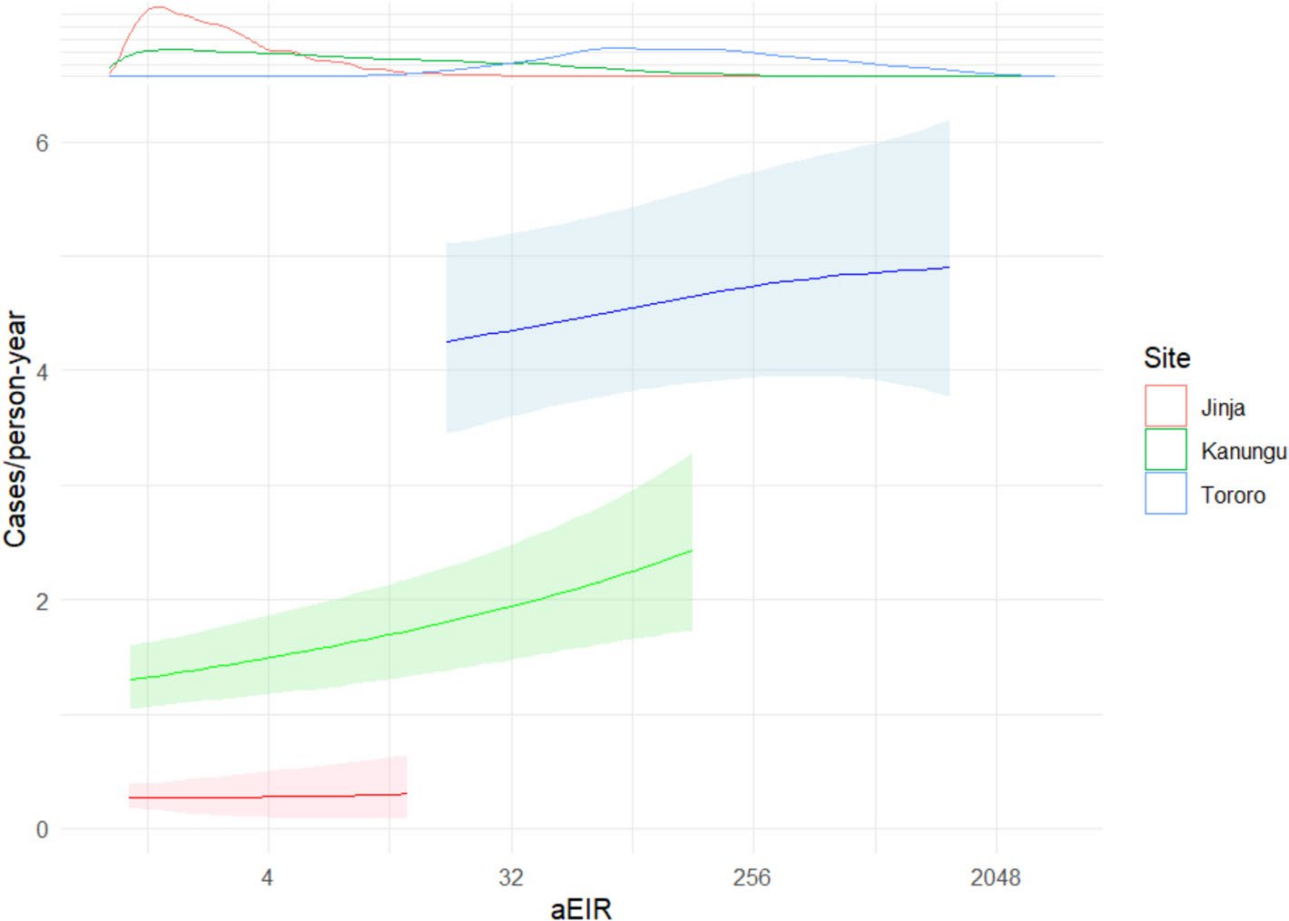
Individual malaria incidence as a smooth function of modeled aEIR with a 14-day lag, grouped by site. Lines show means and ribbons show the 2.5% and 97.5% quantiles of the expected incidence. The density plot at the upper margin indicates the distribution of expected modeled aEIRs by site. The range of predictions for each site is restricted to the 2.5% and 97.5% quantiles of the expected EIRs for that site

To investigate whether the relationship observed when pooling data from all sites applied within sites, models of associations between modeled aEIR and incidence were fit separately for each site (Fig. 3). A positive association between modeled aEIR and incidence was found in Kanungu, but not in Jinja and Tororo. Treating the association between log_2_-transformed spatiotemporally modeled aEIR and incidence as linear on the log scale, on average malaria incidence increased by 9% (IRR 1.09, 95% interval 1.04–1.14) in Kanungu with each doubling of EIR. At both Jinja and Tororo, the 95% credible interval of predicted IRRs for modeled aEIR crossed 1 (Jinja: mean 1.02, 95% interval 0.774–1.26; Tororo: 1.02, 0.986–1.06). Overall, these results were qualitatively similar to trends suggested by average aEIR-incidence plots. In both Kanungu and Tororo, incidence additionally increased with age among younger children before saturating at older ages. No significant relationship was seen in Jinja (Supplementary Fig. 2).

Alternative estimates of aEIR were also investigated, to evaluate whether they could better capture the variance in incidence. Incidence models were fit to crude aEIRs, aEIRs generated from temporal vector count and sporozoite rate smooths (ignoring space), and aEIRs generated from spatial vector count and sporozoite rate smooths (ignoring time), and results were compared by AIC (Supplementary Table 2). The spatiotemporal smooths were the best- or second best-performing models for all sites: in Jinja, the best-performing model by AIC was fit to spatially smoothed aEIRs that ignored time; in Kanungu, models fit to spatiotemporally smoothed aEIRs and temporally smoothed aEIRs that ignored space performed equally well; in Tororo, the best-performing model was fit to temporally smoothed aEIRs. Models fit to crude aEIRs did not converge in Jinja and were the worst-performing by AIC at the other two sites. Regardless of the aEIR estimate used, all models were consistent with a positive association in Kanungu (with the exception of the poorly fitting spatial-only model) but showed no or little association in Tororo and Jinja (Supplementary Figs. 3–4). All models explained a relatively small percentage of the deviance, implying factors not represented in entomological surveillance data influenced malaria incidence [among best-performing models: in Jinja 16.8–21.0%; in Kanungu 9.56–11.6%; and in Tororo 5.48–6.60% (Supplementary Table 2)].

## Discussion

Modeling based on data from clinical surveillance and entomological measures derived from light trap captures, relationships between household aEIR and individual malaria incidence varied between three study sites in Uganda where transmission varies 100-fold. Aggregated results for all sites initially suggested a positive association between aEIR and malaria risk. Analyses stratified by site, however, showed that higher household aEIRs were associated with increasing individual malaria incidence only in Kanungu, an intermediate-EIR and -malaria incidence site. In Jinja (low EIR and low incidence) and Tororo (high EIR and high incidence), associations were weak or absent. There are several possible explanations for this potentially counterintuitive result.

First, previous studies have suggested an underlying sigmoid relationship between EIR and malaria risk [5, 7], which is intuitively appealing: regardless of the exact shape of the EIR-incidence association, there is a maximum number of malaria episodes an individual can experience in a year. In keeping with this pattern, the positive association between EIR and incidence observed at the intermediate-EIR site might correspond to the steep part of an EIR-incidence curve, while the absent associations at the low- and high-EIR sites might correspond to the behaviour of the curve near its minimum and carrying capacity, respectively. This explanation revives the possibility of a general relationship between entomological measures and disease incidence, though it would not account for the lack of overlap between the expected incidences at the three sites as seen in Fig. 3 (implying the existence of at least some site-specific effects).

Second, it is possible that a physical feature unique to Kanungu allowed detection of an association between entomological measures and disease incidence. Kanungu is the only of the three sites with an altitude gradient. This geographical characteristic likely contributes both to a broader within-site aEIR range, across which an association between entomological metrics and incidence may be more easily captured, and to stronger seasonal and spatial trends of mosquito exposure that more closely correlate with corresponding trends in clinical malaria incidence. In this setting, information borrowed from nearby houses and dates would be more informative than at other sites, possibly decreasing variance in the estimates generated by this study.

Third, it is clear from the small proportion of variance explained by the incidence models in this study that there were important drivers of malaria incidence not reflected by entomological surveillance data. Potential candidates include variations in human behaviour, immunity, and mosquito feeding patterns.

Differences in behaviour may mean that household aEIR measured in Jinja and Tororo did not reflect household members’ exposure to infected mosquitoes. This relationship may vary with both local control measures and human behavioural patterns. Imported cases are one particularly important contributor to local malaria incidence: while there is no available direct estimate of the number of imported cases at each site, recent overnight travel has been associated with increased malaria risk at all three sites. Notably, travel was found to be more common in Jinja and Tororo than in Kanungu [18]. At the time of the study, there were no control measures specifically targeted to travelers beyond those available to the general public. LLIN adherence may similarly decouple household EIR from incidence, although bed net distribution was previously shown to have only a modest effect on malaria risk at the study sites despite high reported rates of adherence [11].

Anti-parasite and/or anti-disease immunity is also likely to drive incidence patterns, particularly in the high-transmission setting of Tororo where increasing EIR would be associated with faster development of immunity [19]. While this analysis attempted to minimize the impact of such immunity by restricting the age of the study population to those under five and controlling for the effect of age in the final analysis, these approaches are imperfect. The nonlinear relationship recovered between age and malaria incidence in Kanungu and Tororo is consistent with prior analyses of children under five years in this cohort [19]. Taking advantage of molecular methods to identify incident infections, rather than incident disease, may address some of these concerns by accounting for asymptomatic infections, including superinfections [20].

Finally, local mosquito feeding behaviour may also have differed in ways not captured, or captured differentially by site, in indoor CDC LT data, including variation in baseline biting time and location relative to human behaviours and behavioural plasticity in response to LLIN use. These differences could conceivably stem from environmental heterogeneity, or from differences in species composition within the *An. gambiae* species complex. The variation could potentially have substantial influence on EIR: both *An. gambiae s.s.* and *An. arabiensis* are endemic to the study sites, with *An. arabiensis* exhibiting less anthropophilic and endophagic tendencies [21, 22] and a recent temporal association with lower malaria risk in Tororo [23]. Prior analysis of a subset of mosquito captures from the same cohort suggests that *An. arabiensis* accounts for a substantially smaller proportion of malaria vectors in Kanungu than at the other two sites [18].

The lack of association between entomological and clinical metrics at two of the sites in this study may also reflect the imprecision of entomological data derived from a single CDC LT per household-month, with sporozoite rates estimated from an even smaller subset. As demonstrated by the small percentage of the deviance explained by the sporozoite rate models in particular, the small household-level sample sizes and zero inflation endemic to sporozoite rate measurement remain a challenge for statistical modelling. It may not be possible to fully overcome concerns about the sporozoite rate sample size using the surveillance method employed here, which uses a single trap per household, even if a future study were to test a larger subset of each mosquito capture. Possible alternative approaches for future studies might include placing multiple traps per house or sampling multiple houses in a single cluster or neighborhood. However, it is encouraging that alternative analyses using temporal- or spatial-only models of the sporozoite rate yielded largely consistent results.

Whether CDC LT data themselves are problematic is unclear: EIRs derived from CDC LT data have been comparable with gold standard human-landing catches both in prior analyses of PRISM 1 cohort data and in subsequent entomological studies conducted in Tororo [13, 24], but other studies comparing CDC LT and human-landing catches of anophelines have noted significant differences in overall vector densities, species composition, sporozoite rates, and parous rates [25–28].

To summarize, this study’s use of concurrent longitudinal spatiotemporal entomological and clinical data across a wide range of transmission intensities afforded a uniquely detailed view of the relationship between these two markers of exposure. Nevertheless, the relative lack of overlap in EIRs between the three sites limited the ability of the study to distinguish between potential explanations for the resulting EIR-incidence pattern: either a general, potentially sigmoid, EIR-incidence relationship, or site-specific differences in exposure patterns, host immunity, and/or vector characteristics. Limitations of the study included an inability to characterize direct mosquito-human exposure, as would be afforded by simultaneous human behavioural observations and human-landing catches, a focus on malaria incidence rather than incident *P. falciparum* infection, and a lack of sub-species complex mosquito species identification that might have obscured significant differences in the relative roles played by vector species. Entomological data collections are inherently noisy and sparse relative to the exposure patterns they are meant to reflect, and a fine-scale EIR-incidence association may not exist except in the highly favourable setting offered by a site like Kanungu.

## Conclusions

In conclusion, despite strong theoretical support for a general relationship between the aEIR and malaria incidence, household-level EIRs estimated from smoothed mosquito surveillance data were significantly associated with individual malaria incidences in an intermediate-transmission site, but not at low- or high-transmission sites. Further assessment of this relationship using data collected at a finer temporal scale with molecular identification of new infections may be helpful to tease apart this heterogeneity.

## Supplementary Information

Supplementary material 1.

## Data Availability

The datasets used and/or analysed during the current study are publicly available at ClinEpiDB: https://clinepidb.org/ce/app/workspace/analyses/DS/_0ad509829e.

## Abbreviations

aEIR: Annualized entomological inoculation rate
AIC: Akaike information criterion
CDC LT: CDC light trap
EIR: Entomological inoculation rate
ELISA: Enzyme-linked immunosorbent assay
GAM: Generalized additive model
GAMM: Generalized additive mixed model
IQR: Interquartile range
IRR: Incidence rate ratio
IRS: Indoor residual spraying
LLIN: Long-lasting insecticidal net
PRISM: Program for Resistance, Immunology, Surveillance and Modeling of Malaria
