# Supplementary material for: Relating household entomological measures to individual malaria risk
Source: Malar J. 2025 Aug 14;24:261. doi: 10.1186/s12936-025-05504-5 (PMC12352003; doi:10.1186/s12936-025-05504-5)
Supplement: Supplementary file 1 — Supplementary material 1. [file 12936_2025_5504_MOESM1_ESM.docx]

Supplementary material:

Supplementary Tables 1a-d – Entomological model comparisons

1a - Comparison of candidate vector count generalized additive models. Model family is abbreviated to “Poi” for Poisson, “NB” for negative binomial, and “Tw” for Tweedie. Spatial smooth basis is abbreviated to “TPS” for thin plate spline and “GP” for low-rank gaussian process smooth. Models are compared using AIC and percentage of deviance explained. Additional model diagnostics include dispersion parameter and test and quantile test as provided by the DHARMa package in R.^26^ All models use independent spatial and temporal smooths. Best fitting models for each site are bolded.

| Vector counts | |  |  |  |  | |
| --- | --- | --- | --- | --- | --- | --- |
|  |  |  |  |  |  | |
| Site | **Family** | **Spatial basis** | **AIC** | **% Deviance explained** | | **Diagnostics** |
| Jinja | Poi | TPS | 12391.14 | 53.8 | dispersion 7.2389 (p<0.01); quantile p<0.01 | |
|  |  | GP | 12391.88 | 53.7 | dispersion 7.2212 (p<0.01); quantile p<0.01 | |
|  | NB | TPS | 10158.92 | 43.8 | dispersion 1.3496 (p=0.232); quantile p=0.03361 | |
|  |  | **GP** | **10119.08** | **45.0** | **dispersion 1.3537 (p=0.24); quantile p=0.011** | |
|  | Tw | TPS | 10444.57 | 48.4 | dispersion 2.9575 (p<0.01); quantile p<0.01 | |
|  |  | GP | 10410.15 | 49.1 | dispersion 3.011 (p<0.01); quantile p<0.01 | |
| Kanungu | Poi | TPS | 30421.1 | 64.2 | dispersion 20.774 (p<0.01); quantile p<0.01 | |
|  |  | GP | 30331.4 | 64.3 | dispersion 20.811 (p<0.01); quantile p<0.01 | |
|  | NB | TPS | 17212.75 | 60.3 | dispersion 0.77873 (p=0.52); quantile p<0.01 | |
|  |  | **GP** | **17191.61** | **60.6** | **dispersion 0.78421 (p=0.352); quantile p<0.01** | |
|  | Tw | TPS | 18078.11 | 60.5 | dispersion 2.6104 (p<0.01); quantile p<0.01 | |
|  |  | GP | 18055.16 | 60.8 | dispersion 2.6118 (p<0.01); quantile p<0.01 | |
| Tororo | Poi | TPS | 93910.22 | 69.8 | dispersion 51.687 (p<0.01); quantile p<0.01 | |
|  |  | GP | 93973.84 | 69.8 | dispersion 51.519 (p<0.01); quantile p<0.01 | |
|  | NB | **TPS** | **29058.76** | **57.4** | **dispersion 0.53173 (p<0.01); quantile p<0.01** | |
|  |  | GP | 29077.28 | 57.2 | dispersion 0.54052 (p<0.01); quantile p<0.01 | |
|  | Tw | TPS | 29203.13 | 63.1 | dispersion 1.4991 (p<0.01); quantile p<0.01 | |
|  |  | GP | 29238.58 | 62.8 | dispersion 1.4973 (p<0.01); quantile p<0.01 | |

1b - Comparison of candidate sporozoite rate binomial generalized additive models. Abbreviations as above. All models use independent spatial and temporal smooths. Diagnostics performed on subset of data where number of mosquitoes tested > 0.

| Sporozoite rates | |  |  |  |
| --- | --- | --- | --- | --- |
|  |  |  |  |  |
| Site | **Spatial basis** | **AIC** | **% Deviance explained** | **Diagnostics** |
| Jinja | **TPS** | **351.8285** | **1.82** | **dispersion 0.98997 (p=0.5791); quantile p=0.5791** |
|  | GP | 351.8292 | 1.82 | dispersion 0.99006 (p=0.912); quantile p=0.5797 |
| Kanungu | TPS | 1372.143 | 10.2 | dispersion 0.99112 (p=0.984); quantile p=0.1522 |
|  | **GP** | **1371.037** | **11.1** | **dispersion 0.99387 (p=0.92); quantile p=0.09729** |
| Tororo | TPS | 4761.154 | 10.6 | dispersion 1.2757 (p<0.01); quantile p=0.27 |
|  | **GP** | **4757.526** | **11.8** | **dispersion 1.2734 (p<0.01); quantile p=0.181** |

1c - Comparison of spatial, temporal and spatiotemporal versions of the best-performing vector count generalized additive models. Abbreviations as above.

| Vector counts | |  |  |  |  |
| --- | --- | --- | --- | --- | --- |
|  |  |  |  |  |  |
| Site | **Spatial** | **Temporal** | **AIC** | **% Deviance explained** | **Diagnostics** |
| Jinja | **x** | **x** | **10119.08** | **45.0** | **dispersion NS; quantile p=0.01106** |
|  |  | x | 11132.77 | 19.3 | dispersion 1.581 (p=0.032); quantile NS |
|  | x |  | 10782.32 | 29.8 | dispersion 1.7735 (p<0.01); quantile NS |
| Kanungu | **x** | **x** | **17191.61** | **60.6** | **dispersion NS; quantile p<0.01** |
|  |  | x | 19669.02 | 23.3 | dispersion NS; quantile p<0.01 |
|  | x |  | 18936.68 | 36.9 | dispersion 1.4911 (p=0.008); quantile p<0.01 |
| Tororo | **x** | **x** | **29058.76** | **57.4** | **dispersion 0.5266 (p<0.01); quantile p<0.01** |
|  |  | x | 30113.25 | 42.3 | dispersion 0.71787 (p<0.01); quantile p<0.01 |
|  | x |  | 32118.84 | 10.0 | dispersion 1.2728 (p=0.016); quantile p<0.01 |

1d - Comparison of spatial, temporal and spatiotemporal versions of the best-performing sporozoite rate generalized additive models. Abbreviations as above.

| Sporozoite rates | |  |  |  |  |
| --- | --- | --- | --- | --- | --- |
|  |  |  |  |  |  |
| Site | **Spatial?** | **Temporal?** | **AIC** | **% Deviance explained** | **Diagnostics** |
| Jinja | x | x | 351.8285 | 1.82 | dispersion N; quantile NS |
|  |  | **x** | **350.7768** | **0.92** | **dispersion NS; quantile NS** |
|  | x |  | 350.7826 | 1.20 | dispersion NS; quantile NS |
| Kanungu | **x** | **x** | **1371.037** | **11.1** | **dispersion NS; quantile NS** |
|  |  | x | 1376.046 | 8.00 | dispersion NS; quantile NS |
|  | x |  | 1410.258 | 3.10 | dispersion NS; quantile NS |
| Tororo | **x** | **x** | **4757.526** | **11.8** | **dispersion 1.2758 (p<0.01); quantile NS** |
|  |  | x | 4780.648 | 9.15 | dispersion 1.2871 (p<0.01); quantile NS |
|  | x |  | 4967.879 | 3.47 | dispersion 1.2882 (p<0.01); quantile p=0.01027 |

Supplementary Table 2 - Comparison of AIC and percentage of deviance explained for site-specific incidence models fit to spatiotemporal, spatial and temporal smooths of aEIR, as well as to crude aEIR. Best models by AIC are bolded.

| Site | aEIR smoothing | AIC | % Deviance explained |
| --- | --- | --- | --- |
| Jinja | Spatiotemporal | 1293.0 | 16.9 |
|  | Spatial | **1285.7** | 21.0 |
|  | Temporal | 1296.4 | 16.8 |
|  | Crude | NA | NA |
| Kanungu | Spatiotemporal | **6850.3** | 10.9 |
|  | Spatial | 6936.4 | 9.23 |
|  | Temporal | **6850.3** | 11.6 |
|  | Crude | 84445.0 | 9.56 |
| Tororo | Spatiotemporal | 7328.1 | 6.60 |
|  | Spatial | 7345.2 | 5.81 |
|  | Temporal | **7321.5** | 6.51 |
|  | Crude | 9533.2 | 5.48 |


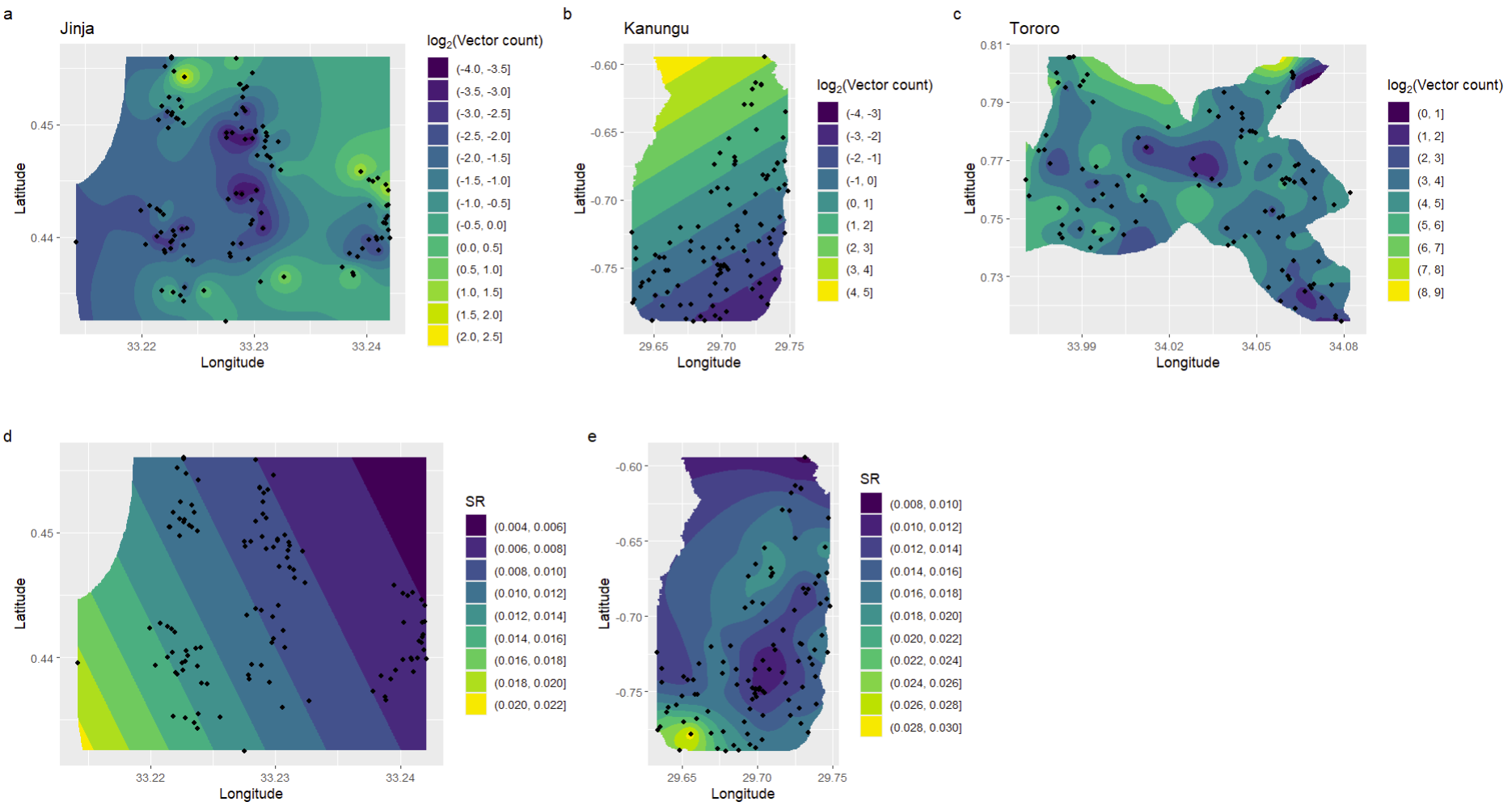


Supplementary Fig. 1 – Filled contour plots of spatial vector count (a-c) and sporozoite rate (d-e) predictions by site, excluding temporal smooths, as generated by the best-fitting entomological generalized additive models. Plots are bounded by subcounty boundaries or latitude and longitude maxima and minima, whichever is more restrictive. Points indicate locations of participating households. No map for sporozoite rate predictions in Tororo is shown because the best-fitting model did not include a spatial smooth.


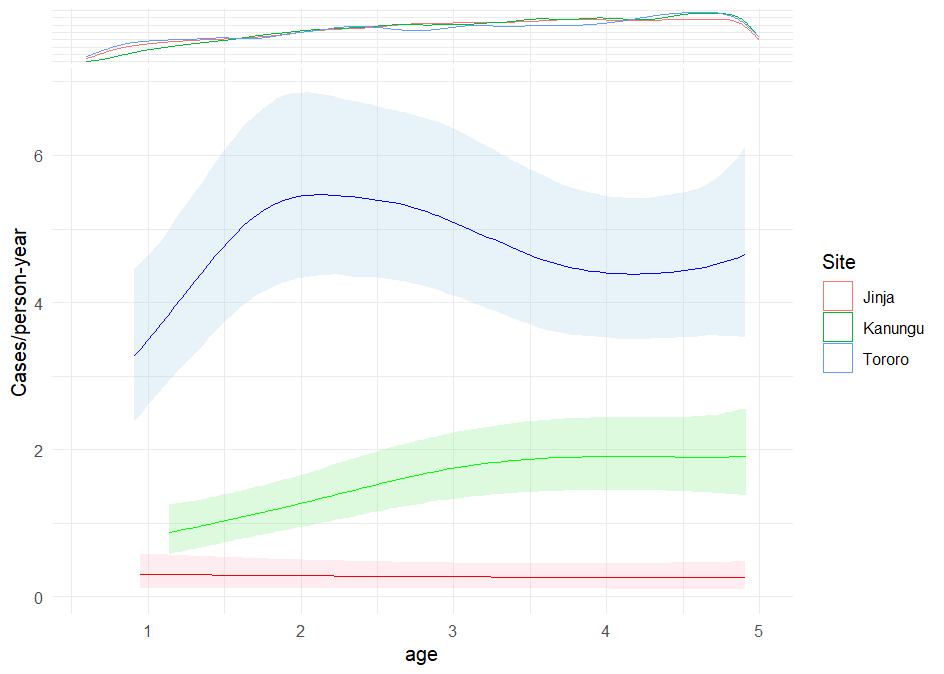


Supplementary Fig. 2 – Individual malaria incidence as a smooth function of age, grouped by site. Ribbons show the 2.5% and 97.5% quantiles of the expected incidence, as predicted by model fit to draws from the prediction interval of the modeled aEIR. The marginal density plot indicates the distribution of ages by site. The extent of each site’s smooth is restricted to the 2.5% and 97.5% quantiles of the ages for that site.


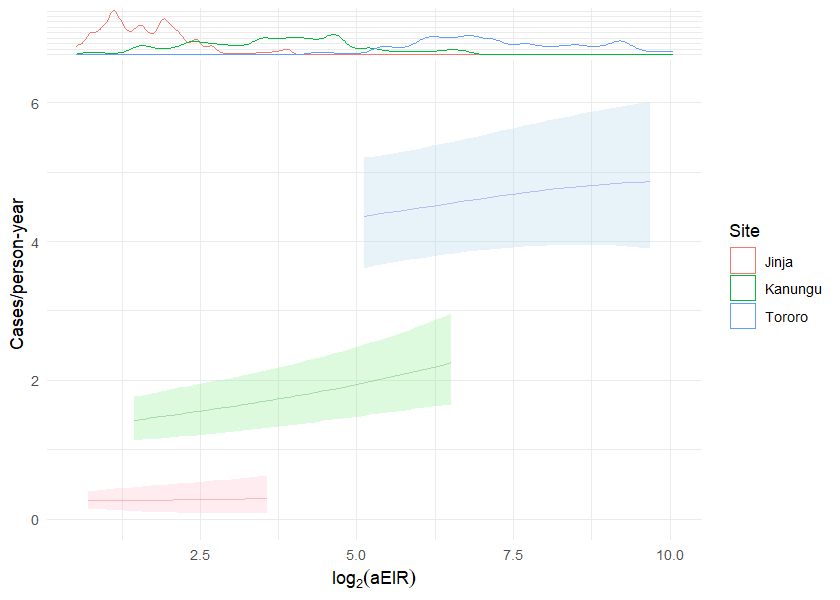


Supplementary Fig. 3 – Individual malaria incidence as a smooth function of temporally smoothed aEIR (no spatial component) with a 14-day lag, grouped by site. Lines show means and ribbons show the 2.5% and 97.5% quantiles of the expected incidence. The density plot at the upper margin indicates the distribution of expected modeled aEIRs. The range of prediction is restricted to the 2.5% and 97.5% quantiles of the expected EIRs for Tororo.


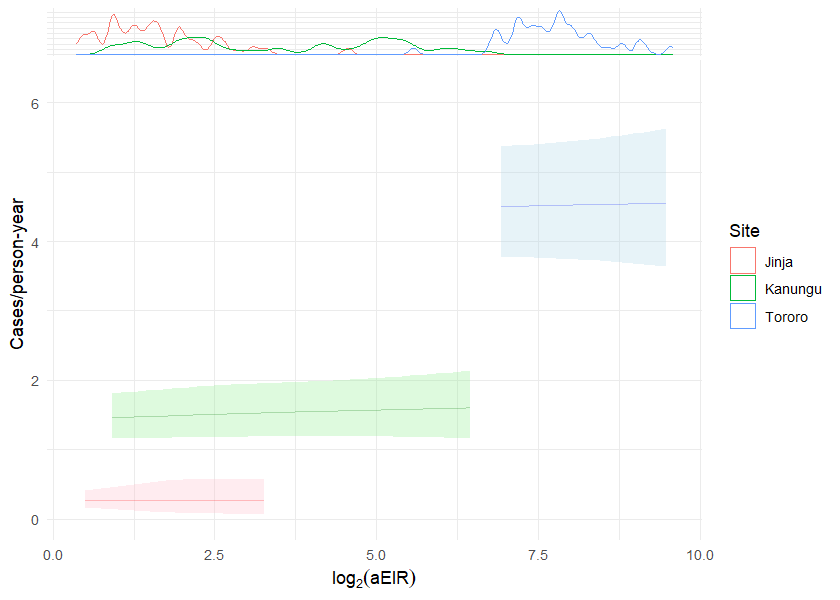


Supplementary Fig. 4 – Individual malaria incidence as a smooth function of spatially smoothed aEIR (no temporal component) with a 14-day lag, grouped by site. Lines show means and ribbons show the 2.5% and 97.5% quantiles of the expected incidence. The density plot at the upper margin indicates the distribution of expected modeled aEIRs. The range of prediction is restricted to the 2.5% and 97.5% quantiles of the expected EIRs for Tororo.
